# Supplementary material for: A general temperature-guided language model to design proteins of enhanced stability and activity
Source: Sci Adv. 2024 Nov 27;10(48):eadr2641. doi: 10.1126/sciadv.adr2641 (PMC11601203; doi:10.1126/sciadv.adr2641)
Supplement: Supplementary file 3 — Erratum published 3 October 2025: In the original Data S1 file, sheet 1 included mislabeled samples due to inadvertent rearrangement of the sample names and numbers during file transfer between different laboratories. Data S1 has been replaced with the corrected version of sheet 1. The authors’ conclusions are not affected by these corrections. The Supplementary Materials PDF and data S1 file have been updated. The original version of the Supplementary Materials PDF is available here: [file sciadv.adr2641_sm.v1.pdf]

Science Advances
AAAS

Supplementary Materials for

A general temperature-guided language model to design proteins of enhanced stability and activity

Fan Jiang *et al.*

Corresponding author: Jie Song, songjie@him.cas.cn; Jia Liu, liujia@shanghaitech.edu.cn; Liang Hong, hongl3liang@sjtu.edu.cn; Pan Tan, tpan1039@gmail.com

Sci. Adv. **10**, eadr2641 (2024)
DOI: 10.1126/sciadv.adr2641

The PDF file includes:

Figs. S1 and S2
Tables S1 to S9
Legend for data S1
References

Other Supplementary Material for this manuscript includes the following:

Data S1

Supplementary Materials

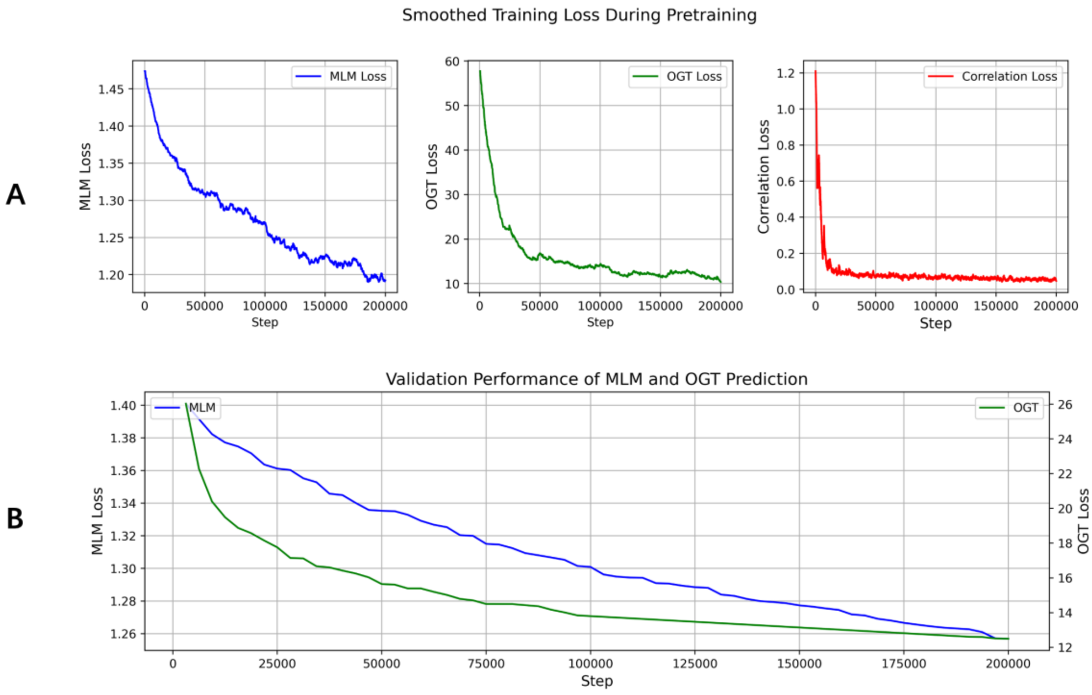

Fig. S1. Training and validation curves during the pre-training process. (A) The smoothed training loss during pre-training. From left to right are MLM Loss, OGT Loss, and Correlation Loss. The stopping criterion for pre-training was reaching the maximum allocated compute budget and we fixed the training steps at 200,000. (B) Validation performance of MLM and OGT predictions. During the pre-training process, we recorded the validation loss on the validation set every 5,000 steps.

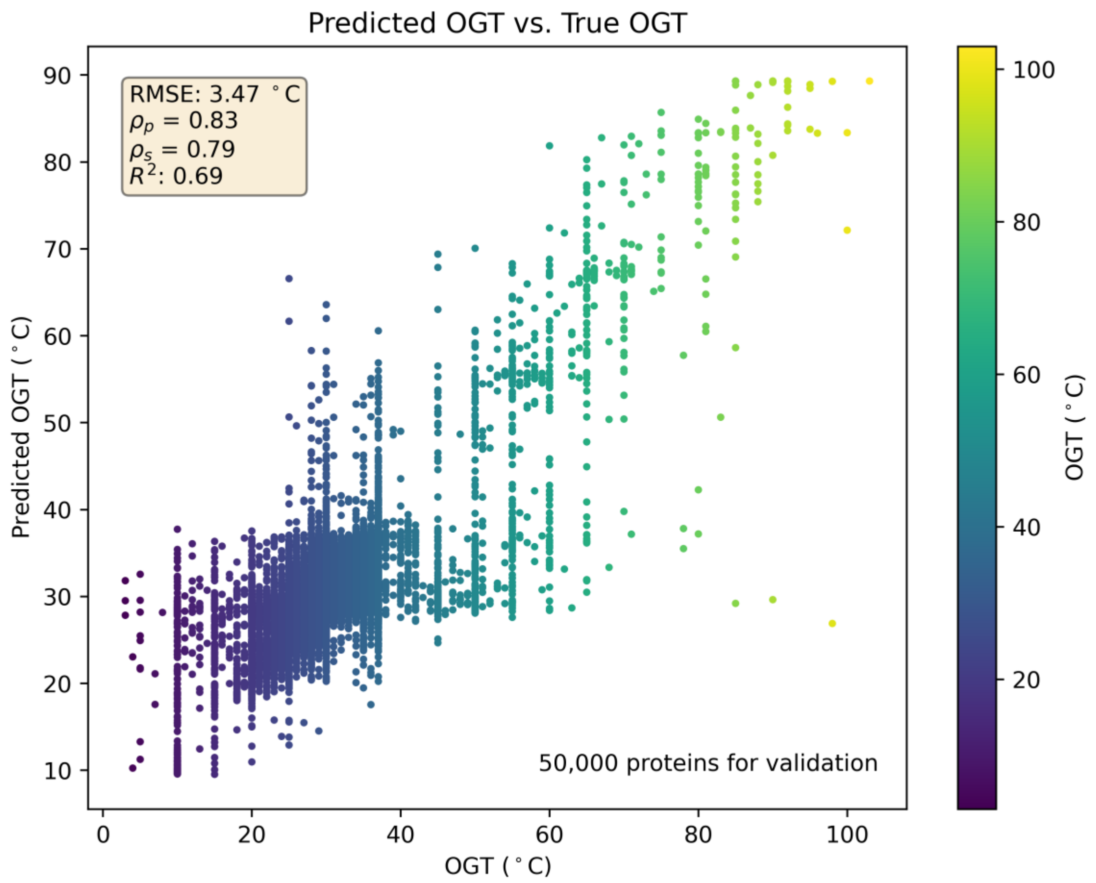

Fig. S2. **Comparison of predicted and actual OGT.** Scatter plot of predicted OGT versus actual OGT on the held-out validation set.

Table S1. Ablation study results for the PRIME model. The numbers in parentheses represent the p-values comparing the baseline PRIME data with the data from the ablation experiments. We used Spearman correlation as our evaluation metric, where a higher value indicates better performance.
PRIME (MSE-Loss) refers to the use of MSE loss to align the MLM and OGT modules. We found that this MSE approach yielded inferior results compared to using Pearson correlation as the loss function.

	ProteinGym	Stability	Activity	ΔT_m
PRIME (-OGT)	0.447 (p=4e-2)	0.564	0.452	0.362 (p=3e-2)
PRIME (-Corr)	0.472 (p=4e-2)	0.588	0.479	0.429 (p=4e-2)
PRIME (-MLM)	0.024 (p=2e-4)	0.130	-0.089	0.150 (p=1e-4)
PRIME (MSE-Loss)	0.432 (p=1e-3)	0.498	0.426	0.363 (p=3e-2)
PRIME	**0.486**	**0.609**	**0.502**	**0.437**

Table S2. Results of benchmarking PRIME against various baseline models on the ProteinGym, Stability, and ΔT_m datasets. The "Stability" dataset refers to ProteinGym-Stability, and "Activity" refers to ProteinGym-Activity. We used Spearman correlation as our evaluation metric, where a higher value indicates better performance. The numbers in parentheses represent the p-values from the Wilcoxon Signed Rank Test, comparing the predictions of the original PRIME model to those of the subsequent models. "PRIME (homo)" denotes the fine-tuning of the PRIME model using the MLM loss on homologous sequences of the target proteins present in either ProteinGym or ΔT_m datasets.

	ProteinGym	Stability	Activity	ΔT_m
ESM-2	0.414 (p=2e-3)	0.523	0.425	0.330 (p=1e-3)
ESM-2 (Homo)	0.438 (p=3e-3)	0.574	0.462	0.385 (p=2e-3)
ESM-1v	0.407 (p=3e-4)	0.477	0.42	0.279 (p=2e-3)
MSA-Transformer	0.434 (p=1e-2)	0.495	0.457	0.327 (p=3e-2)
Tranception-EVE	0.456 (p=2e-3)	0.500	0.487	0.247 (p=8e-3)
SaProt	0.457 (p=1e-4)	0.592	0.458	0.311 (p=1.1e-2)
CARP	0.368 (p=2e-2)	0.412	0.395	0.272 (p=2e-3)
MIFST	0.400 (p=1e-4)	0.485	0.327	0.377 (p=3e-2)
Stability Oracle	0.386 (p=7e-4)	0.568	0.368	0.412 (p=9e-3)
Rosetta	0.154 (p=9.6e-13)	0.154	0.154	0.198 (p=4e-13)
GEMME	0.455 (p=4.1e-2)	0.519	0.484	0.354 (p=2e-2)
PRIME	**0.486**	**0.609**	**0.502**	**0.437**
PRIME (Homo)	0.496 (p=0.039)	0.612	0.521	0.456 (p=8e-3)

Table S3. Supervised prediction of T_m (melting temperature) and T_{opt} (optimal enzymatic activity temperature). Four metrics are employed to gauge the models' accuracy and predictive ability: RMSE, Spearman correlation, Pearson correlation, and R^2 (coefficient of determination). The number in parentheses represents the p-values from the Wilcoxon Signed Rank Test, comparing the predictions of the original PRIME model to those of the subsequent models. We trained PRIME and ESM2 with three different random seeds, while the results for DeepET were obtained from Ref(*82*).

		RMSE	Pearson	R^2	Spearman
T_{opt}	ESM-2-650M(p=1e-12)	13.845(±0.1524)	0.693(±0.012)	0.552(±0.0198)	0.585(±0.0221)
	DeepET(p=1e-9)	12.21	0.76	0.57	0.62
	PRIME	**11.23 (±0.2200)**	**0.81 (±0.0092)**	**0.63 (±0.0142)**	**0.76 (±0.0139)**
T_m		**RMSE**	**Pearson**	**R^2**	**Spearman**
	ESM-2-650M(p=1e-8)	5.589(±0.1642)	0.774(±0.008)	0.592(±0.015)	0.548(±0.02)
	DeepET(p=1e-7)	6.30	0.76	0.58	0.55
	PRIME	**4.80 (±0.1739)**	**0.84 (±0.0064)**	**0.70 (±0.0115)**	**0.64 (±0.0134)**

Table S4. Results from the FLIP benchmark: The results for CARP, ESM-1b and ESM-1v are sourced from the literature (*32*). Spearman correlation is used as the evaluation metric, where a higher value indicates better performance. We implemented a global fine-tuning approach to PRIME and ESM2, where all parameters participated in training.

**AAV**	**mut-des**	**1-vs-many**	**2-vs-many**	**7-vs-many**	**low-vs-high**
CARP-640M	0.750	0.310	0.510	0.580	0.250
ESM-1b	0.700	0.310	0.650	0.610	0.330
ESM-1v	0.790	0.100	0.700	0.700	0.340
ESM-2	0.385	0.440	0.569	0.594	0.310
PRIME	0.82(±0.012)	0.523(±0.01)	0.788(±0.0130	0.762(±0.021)	0.421(±0.013)
**GB1**	**1-vs-many**	**2-vs-many**	**3-vs-many**	**low-vs-high**	
CARP-640M	0.150	0.180	0.620	0.120	
ESM-1b	0.280	0.550	0.790	0.590	
ESM-1v	0.320	0.320	0.770	0.100	
ESM-2	0.180	0.470	0.790	0.530	
PRIME	0.32(±0.008)	0.598(±0.012)	0.824(±0.030)	0.613(±0.014)	
**MELTOME**	**Mixed**	**Human-cell**	**Human**		
CARP-640M	0.530	0.686	0.724		
ESM-1b	0.680	0.750	0.700		
ESM-1v	0.650	0.770	0.780		
ESM-2	0.490	0.627	0.654		
PRIME	0.724(±0.002)	0.825(±0.009)	0.804(±0.023)		

Table S5. The comparative results of four distinct strategies for selecting the top 15 single-point mutations within the ProteinGym dataset, specifically focusing on proteins with saturated single-point mutation data and wild-type sequence similarity below 30% to the PRIME pre-training dataset. The strategies compared are as follows:

1) The strategy method described in this paper, which involves fine-tuning the PRIME model using homologous sequences of the target protein.
2) Fine-tuning ESM2 on the same homologous sequences.
3) The ESM vote strategy from "Hie et al, Nature Biotechnology, 2023,"(*74*), which integrates five ESM-1v models with the ESM1b model, setting alpha to 1 and adjusting the value of *k* for each dataset to yield only 15 mutations.
4) Random mutations, which serve as a baseline for comparison.

The evaluation metrics include the number of positive single-point mutations, the fitness of the most advantageous mutant, and the median fitness of all 15 mutants. The checkpoints files of PRIME (homo) and ESM2 (homo) are available on our GitHub.

Datasets	Metric	PRIME (homo)	ESM2 (homo)	ESM-vote	Random
HSP82_YEAST_Flynn_2019 (0)	Fraction with improved fitness	**7**	4	5	2
	Maximum improved fitness	**0.36**	0.27	0.13	0.11
	Median fitness	**0.02**	-0.12	-0.13	-0.07
KKA2_KLEPN_Melnikov_2014 (1.0)	Fraction with improved fitness	**14**	13	12	2
	Maximum improved fitness	2.48	2.36	**2.75**	1.72
	Median fitness	**1.62**	0.64	1.09	0.75
NKX31_HUMAN_Tsuboyama_2023_2L9R (0)	Fraction with improved fitness	**15**	13	5	2
	Maximum improved fitness	**2.14**	0.76	0.42	0.12
	Median fitness	**0.36**	0.24	-0.16	0.22
PTEN_HUMAN_Mighell_2018 (0)	Fraction with improved fitness	**2**	0	0	0
	Maximum improved fitness	**0.48**	0.00	0.00	0.00
	Median fitness	**-0.84**	-1.06	-0.95	-0.97
TAT_HV1BR_Fernandes_2016 (0)	Fraction with improved fitness	7	5	**8**	2
	Maximum improved fitness	**0.65**	0.24	0.37	0.03
	Median fitness	**0.02**	-0.02	0.01	-0.07

Table S6. The comparative result of the top 15 single-point mutations for T7 RNA polymerase and Tgo-D4K, as selected by four different model methods. The evaluation criteria include the number of positive single-point mutations, the fitness of the most advantageous mutant, and the median fitness of all 15 mutants. For T7 RNA polymerase, fitness is measured by the change in T_m relative to the wild type. For DNA polymerase, fitness is measured by the polymerization activity for 2'-fluoroarabino nucleic acid (a non-natural nucleic acid), with values representing the fold change in activity relative to the wild type. The checkpoints files for PRIME (homo) and ESM2 (homo) strategies are available on our GitHub repository. Rosetta scores protein-saturated single-point mutations by ranking based on predicted values of the unfolding free energy. The energy function used to calculate this unfolding free energy includes all energy terms referenced in the literature (*75*).

Protein	Metric	PRIME (homo)	ESM2 (homo)	ESM-vote	Rosetta
T7 RNA polymerase	Number with improved T_m	**13**	8	7	1
	Maximum improved T_m	**3.5**	0.8	**3.5**	0.6
	Median improved T_m	**1.8**	0.2	0	-0.7
Tgo-D4K	Number with improved fitness	**10**	7	6	5
	Maximum improved fold	**3.27**	2.15	2.07	2.06
	Median fold	**1.21**	0.99	0.93	0.93

Table S7. Results of the in-silico benchmarking of iterative strategies based on PRIME and ftMLDE.

		Zero-Shot	Round 1	Round 2
Max Fitness achieved	Random	0.43 (±0.0389)	0.2397(±0.1612)	0.2286(±0.1577)
	ftMLDE	0.44 (±0.0062)	0.55 (0.0302)	0.55 (0.0820)
	ESM-2 (tiered)	0.45	0.64(±0.02568)	0.66(±0.03645)
	PRIME (tiered)	0.45	0.66(±0.04157)	0.84(±0.0362)
	ESM-2 (top)	0.44	0.66 (±0.0406)	0.66 (±0.0406)
	PRIME (top)	0.45	0.69 (±0.0587)	0.86 (±0.0295)
Mean Fitness achieved	Random	0.06 (±0.0086)	0.01 (±0.0064)	0.01 (±0.0063)
	ftMLDE	0.08 (±0.0034)	0.13 (±0.0018)	0.14 (±0.0017)
	ESM-2 (tiered)	0.07	0.05(±0.0024)	0.1(±0.0011)
	PRIME (tiered)	0.08	0.18(±0.0019)	0.29(±0.0013)
	ESM-2 (top)	0.09	0.07 (±0.0088)	0.10 (±0.0009)
	PRIME (top)	0.11	0.22 (±0.0053)	0.32 (±0.0032)

Table S8. Pseudocode for the alternating training strategy used to optimize MLM, OGT, and Correlation losses.

Input: Mini Batch, training step i.
Output: Loss of the mini-batch.

```
sequence_ids, ogt = batch['sequence_ids'], batch['ogt']
if i % 3 == 1:
    noised_sequence_ids = add_noise(sequence_ids)
    reconstruc_sequence_ids = model(noised_sequence_ids, task = 'MLM')
    loss = compute_mlm_loss(sequence_ids, noised_sequence_ids)
    return loss
elif i % 3 == 2:
    pred_ogt = model(sequence_ids, task = 'OGT')
    loss = mse_loss(pre_ogt, ogt)
    return loss
elif i % 3 == 0:
    reconstruction_logits = model(sequence_ids)
    mutant_indices = random_generate_k_points(sequence_ids)
    mutant_types = random_generate_k_types(sequence_ids)
    mutant_sequence_ids = sequence_ids.clone()
    mutant_sequence_ids.scatter_(1, mutant_indices, mutant_types)
    mlm_scores = reconstruction_logits[:, mutant_indices, mutant_types].log_softmax(dim=-1)
    pred_ogt = prime(mutant_sequence_ids, task = 'OGT')
    loss = 1 - pearson(pred_ogt, mlm_scores)
    return loss
```

Table S9. Performance of zero-shot mutation prediction at a 1:1:1 weight ratio on the ProteinGym and ΔT_m datasets.

MLM Loss Weight	Corr Loss Weight	OGT Loss Weight	ProteinGym	ΔT_m	ProteinGym-Stability
1	1	1	0.440	0.360	0.557
1	0.5	2	0.433	0.351	0.555
0.01	0.5	0.05	0.432	0.349	0.552
1	2	0.05	0.431	0.349	0.551
2	2	2	0.431	0.348	0.550
0.5	2	0.5	0.430	0.348	0.549
1	0.01	0.5	0.430	0.347	0.549
1	1	0.5	0.429	0.346	0.549
0.5	0.5	2	0.429	0.345	0.549
0.5	2	2	0.428	0.345	0.548
0.5	0.5	0.5	0.428	0.345	0.547
1	1	0.01	0.428	0.344	0.547
0.01	1	0.01	0.427	0.344	0.547
0.01	0.5	0.01	0.427	0.344	0.547
0.01	2	0.05	0.427	0.344	0.546
0.05	0.05	2	0.426	0.343	0.545
0.05	1	2	0.426	0.342	0.544
0.5	0.5	1	0.425	0.342	0.544
1	0.05	0.01	0.424	0.342	0.544
0.05	0.01	2	0.424	0.342	0.544
2	2	0.01	0.424	0.341	0.544
0.05	0.05	0.05	0.424	0.341	0.543
0.01	0.01	1	0.424	0.340	0.543
2	2	1	0.423	0.340	0.543
2	0.01	0.05	0.423	0.340	0.542
0.5	0.05	0.5	0.423	0.340	0.542
1	2	1	0.422	0.339	0.542
0.5	1	0.5	0.422	0.339	0.541
0.5	1	1	0.422	0.339	0.541
0.5	0.05	2	0.421	0.339	0.541
0.01	0.05	1	0.421	0.339	0.541
0.05	0.05	0.5	0.420	0.339	0.540
0.05	1	0.05	0.420	0.338	0.540
1	0.01	2	0.420	0.338	0.540
0.01	0.05	0.5	0.419	0.338	0.540
0.5	2	0.01	0.418	0.338	0.540

0.5	0.05	0.05	0.418	0.338	0.540
1	1	0.05	0.418	0.337	0.540
2	0.05	2	0.418	0.337	0.540
0.5	0.5	0.05	0.418	0.337	0.539
0.5	0.01	2	0.418	0.337	0.539
0.5	0.01	0.05	0.418	0.337	0.539
0.5	0.01	0.01	0.418	0.337	0.538
0.05	1	1	0.417	0.337	0.537
0.5	1	0.05	0.417	0.337	0.537
1	0.05	2	0.417	0.336	0.537
0.05	0.5	2	0.417	0.336	0.537
0.05	1	0.01	0.417	0.336	0.537
0.05	0.01	1	0.417	0.336	0.536
0.01	0.5	1	0.416	0.336	0.536
0.05	0.5	0.05	0.416	0.336	0.536
1	0.01	0.01	0.416	0.336	0.536
0.01	2	0.5	0.416	0.335	0.535
0.01	0.5	0.5	0.416	0.334	0.535
0.5	2	1	0.416	0.334	0.535
2	0.5	0.05	0.415	0.334	0.535
0.01	1	1	0.415	0.333	0.535
0.01	0.01	0.01	0.415	0.333	0.534
0.5	0.05	0.01	0.415	0.333	0.534
1	0.5	0.01	0.415	0.332	0.534
0.01	2	2	0.415	0.332	0.534
2	1	2	0.414	0.332	0.534
2	2	0.5	0.414	0.332	0.534
2	0.01	1	0.414	0.332	0.533
2	0.5	0.01	0.414	0.331	0.533
0.5	0.05	1	0.413	0.331	0.533
2	2	0.05	0.413	0.331	0.533
2	0.5	2	0.413	0.331	0.532
2	0.01	0.01	0.412	0.331	0.532
2	0.05	0.05	0.412	0.331	0.532
2	0.5	1	0.412	0.331	0.531
0.05	0.01	0.01	0.412	0.331	0.531
2	1	0.01	0.412	0.330	0.531
1	2	2	0.412	0.330	0.530
0.05	0.5	0.5	0.412	0.330	0.530
0.01	0.01	0.05	0.412	0.330	0.530
1	0.05	1	0.412	0.329	0.530
1	2	0.5	0.411	0.329	0.530

2	1	1	0.411	0.329	0.530
0.01	0.01	2	0.411	0.329	0.529
0.05	2	0.01	0.411	0.329	0.529
0.05	0.05	1	0.411	0.328	0.529
0.05	0.05	0.01	0.411	0.328	0.529
0.05	0.5	1	0.410	0.328	0.529
1	0.01	1	0.410	0.328	0.528
2	0.5	0.5	0.410	0.328	0.528
0.5	0.01	1	0.410	0.327	0.528
0.01	2	0.01	0.410	0.327	0.528
0.05	0.01	0.05	0.410	0.327	0.527
0.5	0.01	0.5	0.410	0.327	0.527
0.05	0.5	0.01	0.410	0.326	0.527
1	0.5	0.5	0.409	0.326	0.527
2	0.05	0.01	0.409	0.326	0.527
0.01	0.05	0.05	0.409	0.325	0.527
1	2	0.01	0.409	0.325	0.526
0.5	2	0.05	0.409	0.324	0.526
1	0.05	0.05	0.409	0.324	0.526
1	0.05	0.5	0.409	0.324	0.526
0.01	1	2	0.408	0.323	0.525
2	0.05	0.5	0.408	0.323	0.525
0.01	0.5	2	0.408	0.323	0.525
0.05	2	1	0.408	0.323	0.525
1	0.5	0.05	0.408	0.323	0.525
0.05	2	0.05	0.407	0.322	0.524
0.05	2	2	0.407	0.322	0.523
0.5	0.5	0.01	0.407	0.322	0.523
0.01	0.01	0.5	0.407	0.322	0.523
0.01	2	1	0.406	0.321	0.523
1	0.5	1	0.406	0.321	0.522
0.05	0.01	0.5	0.406	0.321	0.522
2	1	0.05	0.406	0.320	0.521
0.01	0.05	2	0.406	0.320	0.521
2	1	0.5	0.405	0.320	0.521
0.5	1	2	0.405	0.320	0.521
0.01	1	0.05	0.405	0.319	0.520
2	0.01	0.5	0.405	0.319	0.520
0.01	0.05	0.01	0.402	0.319	0.520
2	0.05	1	0.402	0.318	0.519
0.05	1	0.5	0.401	0.317	0.518
2	0.01	2	0.400	0.316	0.516

0.01	1	0.5	0.399	0.316	0.516
0.05	2	0.5	0.399	0.315	0.514
1	0.01	0.05	0.398	0.312	0.514
0.5	1	0.01	0.397	0.311	0.511
1	1	2	0.390	0.300	0.510

Other Supplementary Material for this manuscript includes the following:

Supplementary Data File: Sheets S1 to S5.

Sheet S1: LbCas12a saturation single-point mutation ranking, featuring single-point mutation T_m results, along with two rounds of multipoint experimental results for T_m and activity.
Sheet S2: T7 RNA polymerase saturation single-point mutation ranking, including single-point mutation T_m results, two rounds of multipoint experimental results for T_m and activity, and the top fifteen single-point mutation types and Tm results predicted by Rosetta, ESM-vote, and ESM2 (homo).
Sheet S3: Creatinase saturation single-point mutation ranking with single-point mutation T_m results.
Sheet S4: Tgo-D4K saturation single-point mutation ranking, including single-point mutation activity results and the top fifteen single-point mutation types and activity results predicted by Rosetta, ESM-vote, and ESM2 (homo).
Sheet S5: VHH saturation single-point mutation ranking, with single-point mutation EC50 results.

REFERENCES AND NOTES

1. W. P. Jencks, *Catalysis in Chemistry and Enzymology* (Courier Corporation, 1987).

2. C. M. O'Connor, J. U. Adams, J. Fairman, *Essentials of Cell Biology* (NPG Education 1, 54, 2010).

3. D. D. Chaplin, Overview of the immune response. *J. Allergy Clin. Immunol.* **125**, S3–S23 (2010).

4. G. B. Cohen, R. Ren, D. Baltimore, Modular binding domains in signal transduction proteins. *Cell* **80**, 237–248 (1995).

5. C. van Vliet, E. C. Thomas, A. Merino-Trigo, R. D. Teasdale, P. A. Gleeson, Intracellular sorting and transport of proteins. *Prog. Biophys. Mol. Biol.* **83**, 1–45 (2003).

6. Y. Xia, X. Li, L. Yang, X. Luo, W. Shen, Y. Cao, L. Peplowski, X. Chen, Development of thermostable sucrose phosphorylase by semi-rational design for efficient biosynthesis of alpha-D-glucosylglycerol. *Appl. Microbiol. Biotechnol.* **105**, 7309–7319 (2021).

7. M. T. Reetz, Z. Sun, G. Qu, *Enzyme Engineering: Selective Catalysts for Applications in Biotechnology, Organic Chemistry, and Life Science* (John Wiley & Sons, 2023).

8. S. L. Lovelock, R. Crawshaw, S. Basler, C. Levy, D. Baker, D. Hilvert, A. P. Green, The road to fully programmable protein catalysis. *Nature* **606**, 49–58 (2022).

9. N. Tokuriki, D. S. Tawfik, Protein dynamism and evolvability. *Science* **324**, 203–207 (2009).

10. S. Lutz, S. M. Iamurri, Protein engineering: Past, present, and future. *Methods Mol. Biol.* **1685**, 1–12 (2018).

11. M. T. Reetz, P. Soni, L. Fernández, Knowledge-guided laboratory evolution of protein thermolability. *Biotechnol. Bioeng.* **102**, 1712–1717 (2009).

12. R. Das, D. Baker, Macromolecular modeling with rosetta. *Annu. Rev. Biochem.* **77**, 363–382 (2008).

13. P. Xiong, Q. Chen, H. Liu, Computational protein design under a given backbone structure with the ABACUS statistical energy function. *Methods Mol. Biol.* **1529**, 217–226 (2017).

14. J. Schymkowitz, J. Borg, F. Stricher, R. Nys, F. Rousseau, L. Serrano, The FoldX web server: An online force field. *Nucleic Acids Res.* **33**, W382–W388 (2005).

15. A. Madani, B. Krause, E. R. Greene, S. Subramanian, B. P. Mohr, J. M. Holton, J. L. Olmos, C. Xiong, Z. Z. Sun, R. Socher, J. S. Fraser, N. Naik, Large language models generate functional protein sequences across diverse families. *Nat. Biotechnol.* **41**, 1099–1106 (2023).

16. B. Hie, D. Zhong Ellen, B. Berger, B. Bryson, Learning the language of viral evolution and escape. *Science* **371**, 284–288 (2021).

17. R. M. Rao, J. Liu, R. Verkuil, J. Meier, J. Canny, P. Abbeel, T. Sercu, A. Rives, MSA transformer, in *Proceedings of the 38th International Conference on Machine Learning* (2021), vol. 139, pp. 8844–8856.

18. A. Rives, J. Meier, T. Sercu, S. Goyal, Z. Lin, J. Liu, D. Guo, M. Ott, C. L. Zitnick, J. Ma, R. Fergus, Biological structure and function emerge from scaling unsupervised learning to 250 million protein sequences. *Proc. Natl. Acad. Sci. U.S.A.* **118**, e2016239118 (2021).

19. Z. Lin, H. Akin, R. Rao, B. Hie, Z. Zhu, W. Lu, N. Smetanin, R. Verkuil, O. Kabeli, Y. Shmueli, A. dos Santos Costa, M. Fazel-Zarandi, T. Sercu, S. Candido, A. Rives, Evolutionary-scale prediction of atomic-level protein structure with a language model. *Science* **379**, 1123–1130 (2023).

20. W. Jin, S. Sarkizova, X. Chen, N. Hacohen, C. Uhler, Unsupervised protein-ligand binding energy prediction via neural Euler's rotation equation. arXiv:2301.10814 [q-bio.BM] (2023).

21. J. Meier, R. Rao, R. Verkuil, J. Liu, T. Sercu, A. Rives, Language models enable zero-shot prediction of the effects of mutations on protein function. bioRxiv 450648 [Preprint] (2021). https://doi.org/10.1101/2021.07.09.450648.

22. C. Hsu, H. Nisonoff, C. Fannjiang, J. Listgarten, Learning protein fitness models from evolutionary and assay-labeled data. *Nat. Biotechnol.* **40**, 1114–1122 (2022).

23. P. Notin, A. Kollasch, D. Ritter, L. Van Niekerk, S. Paul, H. Spinner, N. Rollins, A. Shaw, R. Orenbuch, R. Weitzman, ProteinGym: Large-scale benchmarks for protein fitness prediction and design, in *37th Conference on Neural Information Processing Systems* (NeurIPS 2023).

24. Y. Luo, G. Jiang, T. Yu, Y. Liu, L. Vo, H. Ding, Y. Su, W. W. Qian, H. Zhao, J. Peng, ECNet is an evolutionary context-integrated deep learning framework for protein engineering. *Nat. Commun.* **12**, 5743 (2021).

25. Z. Wu, S. B. J. Kan, D. Lewis Russell, J. Wittmann Bruce, H. Arnold Frances, Machine learning-assisted directed protein evolution with combinatorial libraries. *Proc. Natl. Acad. Sci. U.S.A.* **116**, 8852–8858 (2019).

26. M. K. M. Engqvist, Correlating enzyme annotations with a large set of microbial growth temperatures reveals metabolic adaptations to growth at diverse temperatures. *BMC Microbiol.* **18**, 177 (2018).

27. G. Li, K. S. Rabe, J. Nielsen, M. K. M. Engqvist, Machine learning applied to predicting microorganism growth temperatures and enzyme catalytic optima. *ACS Synth. Biol.* **8**, 1411–1420 (2019) vol 1, pp. 4171–4186.

28. J. Devlin, M.-W. Chang, K. Lee, K. Toutanova, Bert: Pre-training of deep bidirectional transformers for language understanding, in *Proceedings of the 2019 Conference of the North Marican Chapter of the Association for Computational Linguistics: Human Language Technologies,* 4171–4186 (Association for Computational Linguistic, 2019).

29. K. S. Sarkisyan, D. A. Bolotin, M. V. Meer, D. R. Usmanova, A. S. Mishin, G. V. Sharonov, D. N. Ivankov, N. G. Bozhanova, M. S. Baranov, O. Soylemez, N. S. Bogatyreva, P. K. Vlasov, E. S. Egorov, M. D. Logacheva, A. S. Kondrashov, D. M. Chudakov, E. V. Putintseva, I. Z. Mamedov, D. S. Tawfik, K. A. Lukyanov, F. A. Kondrashov, Local fitness landscape of the green fluorescent protein. *Nature* **533**, 397–401 (2016).

30. A. Vaswani, N. Shazeer, N. Parmar, J. Uszkoreit, L. Jones, A. N. Gomez, Ł. Kaiser, I. Polosukhin, Attention is all you need, in *31st Conference on Neural Information Processing Systems* (NIPS 2017).

31. P. Notin, M. Dias, J. Frazer, J. M. Hurtado, A. N. Gomez, D. Marks, Y. Gal, Tranception: Protein fitness prediction with autoregressive transformers and inference-time retrieval, in *Proceedings of the 39th International Conference on Machine Learning* (PMLR, 2022), vol. 162, pp. 16990–17017.

32. K. K. Yang, N. Fusi, A. X. Lu, Convolutions are competitive with transformers for protein sequence pretraining. *Cell Syst.* **15**, 286–294.e2 (2024).

33. K. K. Yang, N. Zanichelli, H. Yeh, Masked inverse folding with sequence transfer for protein representation learning. bioRxiv 493516 [Preprint] (2023). https://doi.org/10.1101/2022.05.25.493516.

34. J. Su, C. Han, Y. Zhou, J. Shan, X. Zhou, F. Yuan, SaProt: Protein language modeling with structure-aware vocabulary. bioRxiv 560349 [Preprint] (2024). https://doi.org/10.1101/2023.10.01.560349.

35. D. J. Diaz, C. Gong, J. Ouyang-Zhang, J. M. Loy, J. Wells, D. Yang, A. D. Ellington, A. G. Dimakis, A. R. Klivans, Stability Oracle: A structure-based graph-transformer framework for identifying stabilizing mutations. *Nat. Commun.* **15**, 6170 (2024).

36. E. Laine, Y. Karami, A. Carbone, GEMME: A simple and fast global epistatic model predicting mutational effects. *Mol. Biol. Evol.* **36**, 2604–2619 (2019).

37. A. Kulandaisamy, R. Sakthivel, M. M. Gromiha, MPTherm: Database for membrane protein thermodynamics for understanding folding and stability. *Brief. Bioinform.* **22**, 2119–2125 (2021).

38. J. Stourac, J. Dubrava, M. Musil, J. Horackova, J. Damborsky, S. Mazurenko, D. Bednar, FireProtDB: Database of manually curated protein stability data. *Nucleic Acids Res.* **49**, D319–D324 (2021).

39. R. Nikam, A. Kulandaisamy, K. Harini, D. Sharma, M. M. Gromiha, ProThermDB: Thermodynamic database for proteins and mutants revisited after 15 years. *Nucleic Acids Res.* **49**, D420–D424 (2021).

40. P. Turner, G. Mamo, E. N. Karlsson, Potential and utilization of thermophiles and thermostable enzymes in biorefining. *Microb. Cell Fact.* **6**, 1–23 (2007).

41. C. Dallago, J. Mou, K. E. Johnston, B. J. Wittmann, N. Bhattacharya, S. Goldman, A. Madani, K. K. Yang, FLIP: Benchmark tasks in fitness landscape inference for proteins. bioRxiv 467890 [Preprint] (2022). https://doi.org/10.1101/2021.11.09.467890.

42. A. Jarzab, N. Kurzawa, T. Hopf, M. Moerch, J. Zecha, N. Leijten, Y. Bian, E. Musiol, M. Maschberger, G. Stoehr, I. Becher, C. Daly, P. Samaras, J. Mergner, B. Spanier, A. Angelov, T. Werner, M. Bantscheff, M. Wilhelm, M. Klingenspor, S. Lemeer, W. Liebl, H. Hahne, M. M. Savitski, B. Kuster, Meltome atlas—Thermal proteome stability across the tree of life. *Nat. Methods* **17**, 495–503 (2020).

43. M. Mirdita, L. Von Den Driesch, C. Galiez, M. J. Martin, J. Söding, M. Steinegger, Uniclust databases of clustered and deeply annotated protein sequences and alignments. *Nucleic Acids Res.* **45**, D170–D176 (2017).

44. Z. Sun, Q. Liu, G. Qu, Y. Feng, M. T. Reetz, Utility of B-factors in protein science: Interpreting rigidity, flexibility, and internal motion and engineering thermostability. *Chem. Rev.* **119**, 1626–1665 (2019).

45. M. Goldsmith, D. S. Tawfik, Enzyme engineering: Reaching the maximal catalytic efficiency peak. *Curr. Opin. Struct. Biol.* **47**, 140–150 (2017).

46. I. Fonfara, H. Richter, M. Bratovic, A. Le Rhun, E. Charpentier, The CRISPR-associated DNA-cleaving enzyme Cpf1 also processes precursor CRISPR RNA. *Nature* **532**, 517–521 (2016).

47. D. C. Swarts, M. Jinek, Mechanistic insights into the cis- and trans-acting DNase activities of Cas12a. *Mol. Cell* **73**, 589–600.e4 (2019).

48. T. S. Zavvar, Z. Khoshbin, M. Ramezani, M. Alibolandi, K. Abnous, S. M. Taghdisi, CRISPR/Cas-engineered technology: Innovative approach for biosensor development. *Biosens. Bioelectron.* **214**, 114501 (2022).

49. S. Borkotoky, A. Murali, The highly efficient T7 RNA polymerase: A wonder macromolecule in biological realm. *Int. J. Biol. Macromol.* **118**, 49–56 (2018).

50. A. Dousis, K. Ravichandran, E. M. Hobert, M. J. Moore, A. E. Rabideau, An engineered T7 RNA polymerase that produces mRNA free of immunostimulatory byproducts. *Nat. Biotechnol.* **41**, 560–568 (2023).

51. G. Leone, B. van Gemen, C. D. Schoen, H. van Schijndel, F. R. Kramer, Molecular beacon probes combined with amplification by NASBA enable homogeneous, real-time detection of RNA. *Nucleic Acids Res.* **26**, 2150–2155 (1998).

52. Z. Cui, Y. Wang, L. Fang, R. Zheng, X. Huang, X. Liu, G. Zhang, D. Rui, J. Ju, Z. Hu, Novel real-time simultaneous amplification and testing method to accurately and rapidly detect *Mycobacterium tuberculosis* complex. *J. Clin. Microbiol.* **50**, 646–650 (2012).

53. J. Nelson, E. W. Sorensen, S. Mintri, A. E. Rabideau, W. Zheng, G. Besin, N. Khatwani, S. V. Su, E. J. Miracco, W. J. Issa, Impact of mRNA chemistry and manufacturing process on innate immune activation. *Sci. Adv.* **6**, eaaz6893 (2020).

54. M. Z. Wu, H. Asahara, G. Tzertzinis, B. Roy, Synthesis of low immunogenicity RNA with high-temperature in vitro transcription. *RNA* **26**, 345–360 (2020).

55. A. J. Killard, M. R. Smyth, Creatinine biosensors: Principles and designs. *Trends Biotechnol.* **18**, 433–437 (2000).

56. Q. Zhi, P. Kong, J. Zang, Y. Cui, S. Li, P. Li, W. Yi, Y. Wang, A. Chen, C. Hu, Biochemical and molecular characterization of a novel high activity creatine amidinohydrolase from Arthrobacter nicotianae strain 02181. *Process Biochem.* **44**, 460–465 (2009).

57. J. A. Berberich, L. W. Yang, I. Bahar, A. J. Russell, A stable three enzyme creatinine biosensor. 2. Analysis of the impact of silver ions on creatine amidinohydrolase. *Acta Biomater.* **1**, 183–191 (2005).

58. F. Jiang, J. Bian, H. Liu, S. Li, X. Bai, L. Zheng, S. Jin, Z. Liu, G.-Y. Yang, L. Hong, Creatinase: Using increased entropy to improve the activity and thermostability. *J. Phys. Chem. B* **127**, 2671–2682 (2023).

59. C. J. Bult, O. White, G. J. Olsen, L. Zhou, R. D. Fleischmann, G. G. Sutton, J. A. Blake, L. M. FitzGerald, R. A. Clayton, J. D. Gocayne, A. R. Kerlavage, B. A. Dougherty, J.-F. Tomb, M. D. Adams, C. I. Reich, R. Overbeek, E. F. Kirkness, K. G. Weinstock, J. M. Merrick, A. Glodek, J. L. Scott, N. S. M. Geoghagen, J. F. Weidman, J. L. Fuhrmann, D. Nguyen, T. R. Utterback, J. M. Kelley, J. D. Peterson, P. W. Sadow, M. C. Hanna, M. D. Cotton, K. M. Roberts, M. A. Hurst, B. P. Kaine, M. Borodovsky, H.-P. Klenk, C. M. Fraser, H. O. Smith, C. R. Woese, J. C. Venter, Complete genome sequence of the methanogenic archaeon. *Science* **273**, 1058–1073 (1996).

60. P. Kois, Z. Tocik, M. Spassova, W.-Y. Ren, I. Rosenberg, J. F. Soler, K. A. Watanabe, Synthesis and some properties of modified oligonucleotides. II. Oligonucleotides containing 2′-deoxy-2′-fluoro-β-D-arabinofuranosyl pyrimidine nucleosides. *Nucleosides Nucleotides* **12**, 1093–1109 (1993).

61. Y. Wang, A. K. Ngor, A. Nikoomanzar, J. C. Chaput, Evolution of a general RNA-cleaving FANA enzyme. *Nat. Commun.* **9**, 5067 (2018).

62. A. Nikoomanzar, M. R. Dunn, J. C. Chaput, Evaluating the rate and substrate specificity of laboratory evolved XNA polymerases. *Anal. Chem.* **89**, 12622–12625 (2017).

63. S. Yan, X. Li, P. Zhang, Y. Wang, H.-Y. Chen, S. Huang, H. Yu, Direct sequencing of 2′-deoxy-2′-fluoroarabinonucleic acid (FANA) using nanopore-induced phase-shift sequencing (NIPSS). *Chem. Sci.* **10**, 3110–3117 (2019).

64. R. P. Harshe, A. Xie, M. Vuerich, L. A. Frank, B. Gromova, H. Zhang, R. J. Robles, S. Mukherjee, E. Csizmadia, E. Kokkotou, A. S. Cheifetz, A. C. Moss, S. K. Kota, S. C. Robson, M. S. Longhi, Endogenous antisense RNA curbs CD39 expression in Crohn's disease. *Nat. Commun.* **11**, 5894 (2020).

65. N. Pelisch, J. Rosas Almanza, K. E. Stehlik, B. V. Aperi, A. Kroner, Use of a self-delivering anti-CCL3 FANA oligonucleotide as an innovative approach to target inflammation after spinal cord injury. *eNeuro* **8**, ENEURO.0338-0320.2021 (2021).

66. V. B. Pinheiro, A. I. Taylor, C. Cozens, M. Abramov, M. Renders, S. Zhang, J. C. Chaput, J. Wengel, S.-Y. Peak-Chew, S. H. McLaughlin, P. Herdewijn, P. Holliger, Synthetic genetic polymers capable of heredity and evolution. *Science* **336**, 341–344 (2012).

67. H. Tang, Y. Gao, J. Han, Application progress of the single domain antibody in medicine. *Int. J. Mol. Sci.* **24**, 4176 (2023).

68. S. Muyldermans, Applications of nanobodies. *Annu. Rev. Anim. Biosci.* **9**, 401–421 (2021).

69. J. Wang, C. R. Bever, Z. Majkova, J. E. Dechant, J. Yang, S. J. Gee, T. Xu, B. D. Hammock, Heterologous antigen selection of camelid heavy chain single domain antibodies against tetrabromobisphenol A. *Anal. Chem.* **86**, 8296–8302 (2014).

70. I. Zettl, T. Ivanova, M. Zghaebi, M. V. Rutovskaya, I. Ellinger, O. Goryainova, J. Kollárová, S. Villazala-Merino, C. Lupinek, C. Weichwald, A. Drescher, J. Eckl-Dorna, S. V. Tillib, S. Flicker, Generation of high affinity ICAM-1-specific nanobodies and evaluation of their suitability for allergy treatment. *Front. Immunol.* **13**, 1022418 (2022).

71. T. M. Pabst, M. Wendeler, X. Wang, S. Bezemer, P. Hermans, A. K. Hunter, Camelid V(H) H affinity ligands enable separation of closely related biopharmaceuticals. *Biotechnol. J.* **12**, 1600357 (2017).

72. B. Palmer, K. Angus, L. Taylor, J. Warwicker, J. P. Derrick, Design of stability at extreme alkaline pH in streptococcal protein G. *Biotechnol. J.* **134**, 222–230 (2008).

73. T. M. Laughlin, J. R. Horn, Engineering pH-sensitive single-domain antibodies. *Methods Mol. Biol.* **2446**, 269–298 (2022).

74. B. L. Hie, V. R. Shanker, D. Xu, T. U. J. Bruun, P. A. Weidenbacher, S. Tang, W. Wu, J. E. Pak, P. S. Kim, Efficient evolution of human antibodies from general protein language models. *Nat. Biotechnol.* **42**, 275–283 (2023).

75. R. F. Alford, A. Leaver-Fay, J. R. Jeliazkov, M. J. O'Meara, F. P. DiMaio, H. Park, M. V. Shapovalov, P. D. Renfrew, V. K. Mulligan, K. Kappel, The Rosetta all-atom energy function for macromolecular modeling and design. *J. Chem. Theory Comput.* **13**, 3031–3048 (2017).

76. S. Biswas, G. Khimulya, E. C. Alley, K. M. Esvelt, G. M. Church, Low-N protein engineering with data-efficient deep learning. *Nat. Methods* **18**, 389–396 (2021).

77. B. J. Wittmann, Y. Yue, F. H. Arnold, Informed training set design enables efficient machine learning-assisted directed protein evolution. *Cell Syst.* **12**, 1026–1045.e7 (2021).

78. J. Meier, R. Rao, R. Verkuil, J. Liu, T. Sercu, A. Rives, Language models enable zero-shot prediction of the effects of mutations on protein function. *Adv. Neural Inf. Process. Syst.* **34**, 29287–29303 (2021).

79. L. Richardson, B. Allen, G. Baldi, M. Beracochea, M. L. Bileschi, T. Burdett, J. Burgin, J. Caballero-Pérez, G. Cochrane, L. J. Colwell, T. Curtis, A. Escobar-Zepeda, T. A. Gurbich, V. Kale, A. Korobeynikov, S. Raj, A. B. Rogers, E. Sakharova, S. Sanchez, D. J. Wilkinson, R. D. Finn, MGnify: The microbiome sequence data analysis resource in 2023. *Nucleic Acids Res.* **51**, D753–D759 (2023).

80. M. Steinegger, M. Mirdita, J. Söding, Protein-level assembly increases protein sequence recovery from metagenomic samples manyfold. *Nat. Methods* **16**, 603–606 (2019).

81. M. Steinegger, J. Söding, Clustering huge protein sequence sets in linear time. *Nat. Commun.* **9**, 2542 (2018).

82. G. Li, F. Buric, J. Zrimec, S. Viknander, J. Nielsen, A. Zelezniak, M. K. M. Engqvist, Learning deep representations of enzyme thermal adaptation. *Protein Sci.* **31**, e4480 (2022).

83. A. J. Meyer, D. J. Garry, B. Hall, M. M. Byrom, H. G. McDonald, X. Yang, Y. W. Yin, A. D. Ellington, Transcription yield of fully 2′-modified RNA can be increased by the addition of thermostabilizing mutations to T7 RNA polymerase mutants. *Nucleic Acids Res.* **43**, 7480–7488 (2015).

84. W. Qin, L. Li, F. Yang, S. Wang, G.-Y. Yang, High-throughput iSpinach fluorescent aptamer-based real-time monitoring of in vitro transcription. *Bioresour. Bioprocess.* **9**, 112 (2022).

85. A. Nikoomanzar, M. R. Dunn, J. C. Chaput, Engineered polymerases with altered substrate specificity: Expression and purification. *Curr. Protoc. Nucleic Acid Chem.* **69**, 4.75.1–4.75.20 (2017).

86. H. Lin, W. Zheng, S. Li, Y. Wang, D. Wei, L. Xie, W. Lu, Z. Tian, S. Wang, J. Qu, J. Liu, Internet of medical things-enabled CRISPR diagnostics for rapid detection of SARS-CoV-2 variants of concern. *Front. Microbiol.* **13**, 1070940 (2022).

87. J. Jumper, R. Evans, A. Pritzel, T. Green, M. Figurnov, O. Ronneberger, K. Tunyasuvunakool, R. Bates, A. Žídek, A. Potapenko, A. Bridgland, C. Meyer, S. A. A. Kohl, A. J. Ballard, A. Cowie, B. Romera-Paredes, S. Nikolov, R. Jain, J. Adler, T. Back, S. Petersen, D. Reiman, E. Clancy, M. Zielinski, M. Steinegger, M. Pacholska, T. Berghammer, S. Bodenstein, D. Silver, O. Vinyals, A. W. Senior, K. Kavukcuoglu, P. Kohli, D. Hassabis, Highly accurate protein structure prediction with AlphaFold. *Nature* **596**, 583–589 (2021).
